# The Therapeutic Paradox of Endocannabinoid Immunomodulation: Molecular Mechanisms and Strategic Frameworks

**DOI:** 10.3390/ijms27156626

**Published:** 2026-07-25

**Authors:** Cameron R. Love

**Affiliations:** Center for Advanced Biotechnology and Medicine, Rutgers University, 679 Hoes Lane, Piscataway, NJ 08854, USA; cl1439@cabm.rutgers.edu

**Keywords:** immune modulation, macrophage polarization, neuroinflammation, NF-κB signaling, cAMP signaling pathway, toll-like receptor 4 (TLR4), gut–immune axis, systemic inflammation

## Abstract

The endocannabinoid system (ECS) is increasingly recognized as a central regulator of immune homeostasis, integrating neural, metabolic, and immune signaling to maintain physiological equilibrium. This Perspective examines the “therapeutic paradox” of endocannabinoid immunomodulation, whereby anti-inflammatory and tissue-protective effects are mechanistically linked to transient immunosuppression. Although cannabinoid receptor 2 (CB2) is the primary mediator of immune regulation, growing evidence indicates that cannabinoid receptor 1 (CB1) also contributes to inflammatory control in both the central nervous system and peripheral tissues. Activation of CB2 suppresses inflammatory signaling through Gi/o-mediated inhibition of adenylate cyclase, reduced cyclic adenosine monophosphate (cAMP) signaling, and repression of nuclear factor kappa B (NF-κB)-dependent transcription. While these mechanisms limit pathological inflammation and promote tissue protection, they simultaneously attenuate innate and adaptive immune functions required for effective pathogen clearance. Across neuroinflammatory disorders, inflammatory bowel disease, hepatic injury, sepsis, cancer, and systemic inflammatory syndromes, the ECS shifts immune responses toward resolution at the cost of reduced antimicrobial readiness. We synthesize the molecular mechanisms underlying this therapeutic paradox, including macrophage polarization, lymphocyte reprogramming, and tissue-specific immune adaptations, and discuss strategies for developing endocannabinoid-based therapeutics that preserve anti-inflammatory efficacy while minimizing immunosuppressive liabilities.

## 1. Introduction

The maintenance of physiological life requires a continuous, highly sophisticated calibration between two opposing biological imperatives: the rapid, aggressive elimination of invading infectious agents and the strict, well-timed limitation of collateral tissue damage induced by the host’s own inflammatory responses [1]. The prompt, unhindered initiation of inflammatory cascades is absolutely vital for early host defense, signaling the recruitment of innate effectors to sites of injury or microbial invasion [2]. However, an inability to terminate these programs efficiently results in persistent, self-destructive pathology [3]. When the normal regulatory mechanisms governing the termination of inflammation fail, the resulting homeostatic breakdown drives a broad spectrum of human diseases [4]. These include chronic conditions such as inflammatory bowel disease, progressive neurodegenerative disorders, systemic autoimmune conditions, malignancies where chronic inflammation drives tumor initiation, progression, metastasis, and immune evasion, acute life-threatening syndromes like sterile or infectious sepsis, and the accelerated decline associated with chronic inflammatory aging phenotypes [5,6,7,8]. To manage these complex responses, immune homeostasis is maintained through the coordinated action of multiple parallel regulatory pathways. Among these, the endocannabinoid system (ECS) has emerged as a premier, centrally integrative lipid signaling network dedicated to governing this delicate immunological equilibrium [9,10,11]. Initially characterized within the strict context of neurophysiology, retrograde synaptic transmission, and central psychotropic activity, the ECS is now recognized as a ubiquitous, on-demand lipid signaling architecture present in virtually every tissue type [12,13]. It is composed fundamentally of endogenous lipophilic ligands—primarily N-arachidonoylethanolamine, also known as anandamide (AEA), and 2-arachidonoylglycerol (2-AG); their specialized biosynthetic and degradative metabolic enzymes; and two primary seven-transmembrane G protein-coupled receptors (GPCRs), denoted cannabinoid receptor 1 (CB1) and CB2 [14,15,16]. While CB1 is heavily enriched within the central nervous system, where it acts as a gatekeeper for neurotransmitter release, CB2 is preferentially and densely expressed throughout the peripheral and central immune compartments [17,18]. It is found in exceptionally high abundance on the surfaces of macrophages, neutrophils, B cells, T cells, dendritic cells, and microglia, strongly implying an evolutionary specialization in immunoregulatory architecture [19,20,21].

Extensive pharmacological and genetic studies consistently demonstrate that endocannabinoid signaling effectively suppresses pro-inflammatory cytokine production, reduces leukocyte recruitment and rolling affinity, modulates oxidative stress cascades, and promotes tissue repair pathways across diverse preclinical disease models [22,23,24,25]. However, these beneficial anti-inflammatory effects are not isolated from immune suppression; rather, they emerge from direct interference with canonical immune activation pathways [26,27]. The ECS therefore operates as a physiological counter-regulatory brake that prioritizes tissue preservation over maximal immune aggression [28,29]. This inherent duality introduces a profound therapeutic paradox, wherein the very mechanisms that suppress destructive hyper-inflammation concurrently compromise the host’s immediate antimicrobial readiness [30].

This manuscript is structured as a Perspective article to present an original conceptual framework defining this “therapeutic paradox.” The specific purpose of this review is to explicitly delineate how endocannabinoid immunomodulation is defined not simply as an anti-inflammatory switch but as a state of direct, competitive signaling within the immune system that actively shifts target cells away from aggressive defensive execution and toward metabolic resolution [31]. By formally conceptualizing this phenomenon as a fundamental biological trade-off, this Perspective advances beyond traditional literature reviews that catalog anti-inflammatory benefits, instead offering a novel paradigm where immune suppression is viewed as the integrated, structural cost of enforcing tissue homeostasis. This framework provides a structured lens through which to evaluate the competing intracellular signaling pathways, macrophage polarization shifts, and lymphocyte reprogramming networks discussed in subsequent sections. Furthermore, by distinguishing established signaling configurations from emerging, speculative mechanisms, this synthesis clarifies where the molecular evidence governing this paradox is strongest and where additional investigation is required to safely translate these pathways into targeted clinical therapeutics.

To support the development of this conceptual framework, a targeted methodology was utilized for the literature review. The primary databases searched included PubMed, Scopus, and Web of Science, covering a time period from 2000 to 2026 to capture both foundational discoveries and recent advancements. The search strategy employed Boolean combinations of keywords such as “endocannabinoid system,” “CB2 receptor,” “immune modulation,” “macrophage polarization,” “immunosuppression,” and “inflammation.” Criteria for selecting cited publications required articles to be peer-reviewed, published in English, and specifically focused on molecular signaling cascades, in vivo immunological trade-offs, or targeted preclinical models of disease. Studies strictly isolated to the behavioral or psychotropic effects of cannabinoids without an immunological component were excluded to maintain a rigorous focus on the therapeutic paradox.

## 2. The Endocannabinoid System as an Immunological Regulatory Network

Transitioning from broad physiological regulation to molecular signaling dynamics, the ECS functions as a highly dynamic, localized, lipid-based signaling network wherein ligand synthesis is tightly coupled to cellular stress, mechanical injury, and inflammatory activation [31]. Unlike classical endocrine hormones or peptide neurotransmitters that are pre-synthesized and stored in intracellular vesicles waiting for exocytic release, the major endocannabinoids AEA and 2-AG are synthesized strictly on demand directly from membrane phospholipid precursors in response to elevated intracellular calcium levels and receptor stimulation [32,33]. AEA is liberated from membrane fractions via the actions of N-acylphosphatidylethanolamine-specific phospholipase D, while 2-AG is generated via diacylglycerol lipase isoforms [34]. Once synthesized, these lipid mediators are released into the extracellular space to act locally on adjacent immune cells in a paracrine fashion or on the synthesizing cell itself via autocrine loops. Their actions are kept highly localized due to rapid enzymatic degradation and cellular reuptake, where AEA is hydrolyzed into arachidonic acid and ethanolamine by fatty acid amide hydrolase (FAAH), and 2-AG is primarily broken down by monoacylglycerol lipase (MAGL) [35,36]. This rapid metabolic turnover ensures spatially and temporally restricted immune modulation, localized precisely to the microenvironment of cellular injury or pathogen encounter [37].

At the systems level, the ECS operates in a bidirectional, highly integrated manner within immune compartments [38]. Innate and adaptive immune cells are not merely passive targets of cannabinoid signaling; they actively synthesize, transport, and degrade endocannabinoids, thereby establishing sophisticated autocrine and paracrine feedback loops that continuously tune the basal and active immune tone [24,39]. The enrichment of the CB2 receptor in the spleen, lymph nodes, tonsils, and circulating peripheral immune cells underscores its evolutionary specialization in immune regulation [18,40]. When the immune system is provoked by an external or internal insult, ECS activation modulates a vast array of simultaneous cellular processes, including antigen presentation, pro- and anti-inflammatory cytokine secretion profiles, leukocyte chemotaxis, vascular rolling affinity, NLR family pyrin domain containing 3 (NLRP3) inflammasome activation, oxidative burst generation, apoptosis, and cellular metabolic reprogramming [41,42,43].

Critically, ECS activity and receptor expression are markedly upregulated during inflammatory stress states, such as active inflammatory bowel disease, obesity-associated metabolic inflammation, neurodegenerative diseases, and acute sterile or infectious injuries [44,45,46]. This inducible nature marks the system as a primary, endogenous anti-inflammatory buffering network designed to curtail runaway immune responses and initiate resolution cascades [46,47]. Critically, CB2 gene expression is directly linked to the epigenetic landscape of the cell, showing upregulation during transitions from basal monitoring states to active inflammatory defense states [48]. This context-dependent upregulation acts as a physiological circuit breaker. When pattern recognition receptors or cytokine signals reach a threshold of chronic or hyper-acute activation, the transcriptomic machinery rapidly drives the transcription of the CB2 receptor network to dampen the local inflammatory velocity.

## 3. Molecular Mechanisms of Cannabinoid-Mediated Immune Suppression and Organelle Dynamics

**Figure 1:**
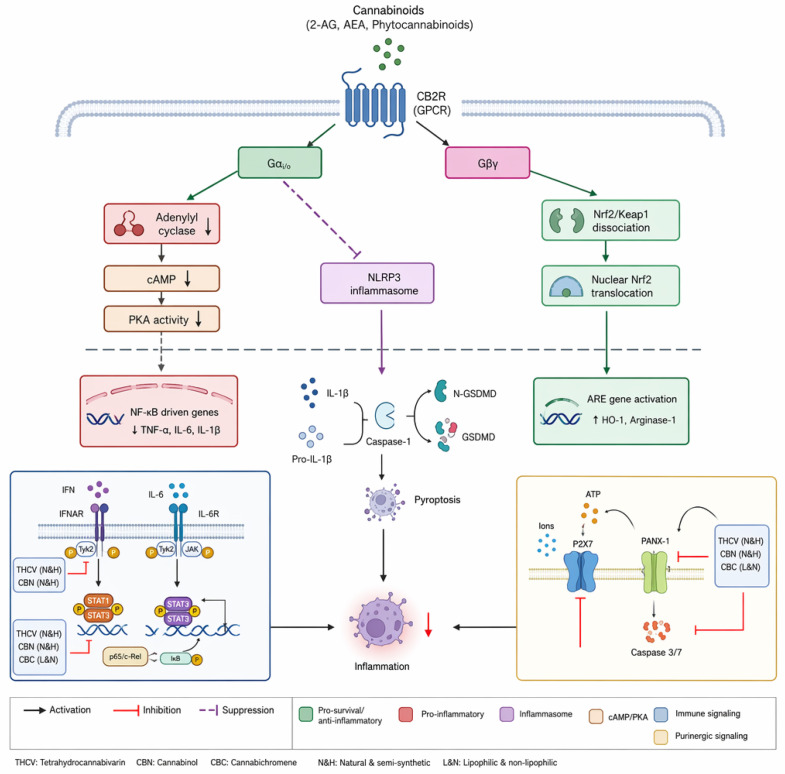
Molecular mechanisms of endocannabinoid-mediated immunomodulation and the “therapeutic paradox.” This figure illustrates the signaling cascades engaged by cannabinoid receptor 2 (CB2R) activation and their role in the therapeutic paradox of immune suppression versus tissue protection. Binding of cannabinoids (e.g., 2-AG, AEA, or phytocannabinoids) to the G protein-coupled receptor (GPCR) CB2R initiates dual signaling through Gαi/o and Gβγ subunits. The Gαi/o pathway leads to the inhibition of adenylyl cyclase, decreasing intracellular cAMP levels and suppressing PKA activity, which results in the downregulation of NF-κB-driven pro-inflammatory genes, including TNF-α, IL-6, and IL-1β. Concurrently, CB2R activation suppresses the NLRP3 inflammasome, limiting the processing of pro-IL-1β and the maturation of caspase-1, thereby mitigating pyroptosis and reducing inflammatory output. The Gβγ signaling axis promotes the dissociation of the Nrf2/Keap1 complex, facilitating the translocation of Nrf2 to the nucleus. This initiates the activation of antioxidant response elements (AREs), leading to the upregulation of cytoprotective genes such as HO-1 and arginase-1. The blue box highlights the modulation of IFN/IL-6 receptor signaling, where specific phytocannabinoids (THCV, CBN, and CBC) inhibit the JAK/STAT and NF-κB pathways. The yellow box outlines the purinergic signaling axis, demonstrating the inhibitory impact of phytocannabinoids on P2X7 and PANX-1 channels and caspase-3/7 activity. Collectively, these mechanisms demonstrate that while cannabinoid signaling suppresses destructive hyper-inflammation, these same pathways compete with and truncate the host’s acute antimicrobial immune defenses, forming the basis of the “therapeutic paradox.” Abbreviations: 2-AG, 2-arachidonoylglycerol; AEA, anandamide; ARE, antioxidant response element; cAMP, cyclic adenosine monophosphate; CBC, cannabichromene; CBN, cannabinol; CB2R, cannabinoid receptor 2; GPCR, G protein-coupled receptor; HO-1, heme oxygenase-1; IFN, interferon; IL-6R, interleukin-6 receptor; JAK, Janus kinase; Keap1, Kelch-like ECH-associated protein 1; NF-κB, nuclear factor kappa B; NLRP3, NLR family pyrin domain containing 3; Nrf2, nuclear factor erythroid 2-related factor 2; PANX-1, pannexin-1; PKA, protein kinase A; STAT, signal transducer and activator of transcription; THCV, tetrahydrocannabivarin; TNF-α, tumor necrosis factor-alpha; Tyk2, tyrosine kinase 2.

The established molecular signature of canonical CB2 receptor signaling is predominantly characterized by its coupling to heterotrimeric Gi/o proteins [49]. Upon ligand binding, the active alpha subunit dissociates from the beta-gamma complex, leading to the direct inhibition of adenylyl cyclase enzymes [50]. This inhibition causes a rapid, sustained drop in intracellular cAMP levels, which subsequently suppresses the activity of protein kinase A (PKA) [51]. The downstream consequence of this pathway is the severe attenuation of NF-κB transcriptional activity [52,53]. Under normal inflammatory activation, such as via Toll-like receptors (TLRs) reacting to microbial pathogen-associated molecular patterns, PKA facilitates the phosphorylation and optimal transcriptional assembly of NF-κB subunits, enabling their nuclear translocation and binding to inflammatory promoters [54]. By decreasing intracellular cAMP and suppressing PKA activity, CB2 signaling can attenuate this activation node, reducing transcription of key inflammatory mediators including tumor necrosis factor-alpha (TNF-α), interleukin-1 beta (IL-1β), interleukin-6 (IL-6), and interferon-gamma (IFN-γ) [55,56,57]. In this regulatory model, CB2 orchestrates a complex dual-signaling network through Gαi/o and Gβγ subunits that serves to both dampen pro-inflammatory transcriptional output and simultaneously induce antioxidant resolution programs (Figure 1). Through the Gαi/o branch, ligand binding directly inhibits adenylyl cyclase, resulting in reduced intracellular cAMP concentrations and attenuated protein kinase A (PKA) activity, which collectively diminish the activation and nuclear translocation of NF-κB, thereby suppressing the expression of pro-inflammatory cytokines such as TNF-α, IL-6, and IL-1β. Furthermore, this pathway impedes NLRP3 inflammasome activation, limiting caspase-1 maturation and pyroptotic cell death. Conversely, the Gβγ signaling axis triggers Nrf2/Keap1 dissociation, enabling Nrf2 to enter the nucleus and stimulate the expression of antioxidant defense genes, such as HO-1 and arginase-1. This signaling architecture also encompasses inhibitory effects on the JAK/STAT pathway and purinergic channels like P2X7 and PANX-1, underscoring how endocannabinoids exert their immunomodulatory effects at multiple convergent signaling nodes (Figure 1).

Crucially, emerging literature hypothesizes that the structural architecture of endocannabinoid immunomodulation may extend beyond classical plasma membrane actions, embedding itself within the regulatory topography of cellular organelles [58,59]. However, these interpretations remain highly speculative, predominantly based on preclinical in vitro models, and warrant clear distinction from established pathways. A fraction of cannabinoid receptors has been observed to localize to internal intracellular membranes, including the outer and inner mitochondrial membranes, the endoplasmic reticulum, and the lysosomal–endosomal degradation compartment [60,61]. Specifically, mitochondrial CB1 (mtCB1) receptors have been structurally identified and functionally linked to the direct regulation of cellular respiration. Activation of these mtCB1 receptors actively suppresses the mitochondrial electron transport chain, reducing oxidative phosphorylation and profoundly shifting the metabolic phenotype of the cell. Concurrently, mitochondrial CB2 (mtCB2) receptors are increasingly recognized as critical regulators of immune cell metabolic reprogramming, acting to modulate intramitochondrial cAMP and govern the bioenergetic shifts necessary for active immune responses. The proposed existence of functional mitochondrial cannabinoid receptors suggests a potential molecular link between endocannabinoid exposure and the regulation of cellular bioenergetics [61,62,63]. Some models argue that activation of mitochondrial cannabinoid receptors might suppress local intramitochondrial AC activity, leading to a localized reduction in cAMP concentrations and PKA activity within the mitochondrial matrix [61]. This proposed intra-organellar signaling axis is theorized to directly dampen electron transport chain efficiency by downregulating Complex I enzymatic activity and inhibiting cellular respiration, which could shift the energy-sensing landscape of the immune cell [63]. While intriguing, this multiphasic control of mitochondrial respiration is a developing framework that requires rigorous in vivo corroboration.

Similarly, interpretations suggest endocannabinoid signaling may influence mitochondrial–endoplasmic reticulum crosstalk, which is required for coordinating cellular stress responses and the mobilization of intracellular calcium pools [58,64]. By theoretically targeting the junctions between the endoplasmic reticulum and adjacent mitochondria, some evidence hints that endocannabinoids regulate calcium oscillations, potentially buffering cells against toxic cytoplasmic calcium overloads during persistent immune challenge [64]. Concurrently, speculative models posit that intracellular endocannabinoid signaling triggers endoplasmic reticulum stress-dependent pathways that activate the unfolded protein response and interface directly with macroautophagic networks [58,65]. Activation of intracellular CB2 receptors is hypothesized to induce a pro-autophagy program that governs macroautophagic vacuole recycling, facilitating the clearance of damaged organelles, misfolded proteins, and aggregated macromolecular structures such as the NLRP3 inflammasome [65]. This theoretical organelle-level control mechanism is thought to function as a metabolic circuit breaker. By enforcing autophagy and modulating the mitochondrial redox state, proponents argue that endocannabinoids actively suppress the generation of mitochondrial reactive oxygen species (ROS), thereby ensuring that downstream inflammasome oligomerization and subsequent IL-1β maturation are post-translationally arrested [25,65]. These sub-cellular mechanisms are further hypothesized to prevent the induction of mitochondrial permeability transition pore opening, stabilizing the inner mitochondrial membrane potential and shielding the cell from pyroptotic or necrotic breakdown [64]. It must be noted, however, that significant questions remain regarding the accuracy of extrapolating these in vitro organelle models to systemic clinical pathology.

Conversely, established pathways clearly show CB2 signaling enhances the expression and secretion of powerful anti-inflammatory and pro-resolving cytokines, most notably interleukin-10 (IL-10) and transforming growth factor-beta (TGF-β), driving cells toward active immune resolution phenotypes [59]. Beyond this canonical cAMP and PKA axis, CB2 activation engages alternative pathways via β-gamma subunits and β-arrestin scaffolding, including mitogen-activated protein kinase and phosphoinositide 3-kinase pathways [66,67]. More recent investigations into non-canonical signaling have also demonstrated that CB2 receptor activation can stimulate transient intracellular calcium fluctuations through phospholipase C mobilization in specialized endothelial and immune subtypes, expanding the diversity of its intracellular fingerprint [48].

A particularly vital and established downstream effector mechanism is the activation of the nuclear factor erythroid 2-related factor 2 (Nrf2) and heme oxygenase-1 (HO-1) signaling axis [68]. CB2 signaling promotes the dissociation of nuclear factor erythroid 2-related factor 2 from its cytosolic inhibitor, facilitating its translocation into the nucleus where it binds to antioxidant response elements (AREs) [69]. This shifts immune cell metabolism heavily toward antioxidant defense, upregulating protective enzymes like heme oxygenase-1 while actively suppressing pro-inflammatory gene expression [70,71]. Natural phytocannabinoids like cannabidiol have been shown to integrate this exact redox-related control framework, downregulating ROS production while simultaneously shutting down redox-sensitive inflammasome components [72].

## 4. Macrophage Polarization as a Central Axis of ECS-Mediated Immune Competition

Macrophages represent a foundational functional node where endocannabinoid signaling pathways directly compete and crosstalk with canonical immune activation signals [73]. These innate immune cells possess remarkable phenotypic plasticity, capable of shifting across a broad activation spectrum ranging from the M1 pro-inflammatory state to the M2 reparative state [74,75]. M1 macrophages are rapidly induced by pathogen-associated molecular patterns, such as lipopolysaccharide, alongside IFN-γ and direct TLR4 activation [76]. Once polarized, M1 cells produce massive amounts of TNF-α, IL-6, inducible nitric oxide synthase, and ROS [77]. While these highly toxic effector molecules are absolutely essential for intracellular pathogen killing and tumor clearance, they act as major drivers of collateral tissue damage when left unchecked [78]. Conversely, M2 macrophages are induced by interleukin-4 and interleukin-13 signaling networks. They are characterized by robust IL-10 production, high arginase-1 expression, extracellular matrix remodeling capacity, angiogenesis promotion, and the active resolution of tissue inflammation [79,80]. CB2 receptor activation actively skews macrophages away from M1 dominance and directly drives them toward an M2 polarization state [81,82]. This is achieved through the dual mechanism of suppressing the TLR4 and NF-κB signaling cascade and enhancing Nrf2-dependent transcriptional programs [83,84].

However, it is crucial to acknowledge that the classic M1/M2 paradigm represents an oversimplified conceptual model. Current immunological understanding recognizes that macrophage activation exists along a highly dynamic and heterogeneous continuous spectrum, driven by complex microenvironmental cues rather than strictly binary phenotypes. While the M1/M2 classification remains a useful heuristic for describing extremes of polarization in vitro, in vivo macrophage populations often exhibit mixed phenotypes that simultaneously blend pro-inflammatory and reparative transcriptional signatures. Endocannabinoids likely act across this entire spectrum to dial down inflammatory intensity rather than forcing an absolute binary switch.

Regardless of the exact position on this polarization spectrum, ECS-mediated macrophage reprogramming serves as the cellular embodiment of the therapeutic paradox. By skewing the cell’s transcriptomic profile toward resolution (M2-like features) at the expense of pathogen-killing machinery (M1-like features), the ECS prioritizes long-term tissue viability over immediate, aggressive pathogen neutralization. Because M1 macrophages and their associated oxidative bursts are strictly required for early-stage antimicrobial defense, endocannabinoid-driven suppression of M1 polarization creates a transient but dangerous window of vulnerability [85]. Preclinical models provide striking evidence of this phenomenon. For instance, cannabinoid receptor activation significantly improves host survival in galactosamine- and lipopolysaccharide-induced acute liver failure models by suppressing destructive M1 inflammatory macrophage activation while simultaneously accelerating the appearance of reparative M2 polarization [86,87]. Similarly, cannabinoid administration in murine models of colitis markedly decreases pro-inflammatory macrophage infiltration, thereby shielding the intestinal mucosa from catastrophic tissue injury [88,89].

This phenotype-reprogramming effect has been confirmed in human cellular translation systems. Clinical observations in pediatric inflammatory bowel disease patients demonstrate that circulating macrophages derived from active Crohn’s disease and ulcerative colitis cases possess significantly reduced basal levels of CB2 receptor expression, which correlates directly with an overrepresented M1 profile and severe mucosal barrier disruption [81,90]. When these patient-derived cells are treated ex vivo with the selective CB2 agonist JWH-133, the receptor network triggers structural M2 polarization, limits the release of toxic cytokines, corrects aberrant macrophage iron metabolism by adjusting ferroportin-1 and hepcidin expressions, and significantly reinforces the structural integrity of the intestinal epithelial monolayer [81,90].

When this system is subjected to co-exposures or multi-toxin microenvironments, competitive receptor interactions alter the homeostatic equilibrium. For example, recent investigations into combined toxicological profiles show that while acute ethanol exposure forces macrophages toward a highly destructive, hyper-inflammatory M1 phenotype accompanied by elevated monocyte chemoattractant protein-1, transforming growth factor-alpha, and TNF-α secretion, the concurrent engagement of cannabinoid pathways via synthetic agonists acts as an antagonistic immunomodulator [91]. In these scenarios, selective pharmacological blockade of CB2 markedly diminishes this buffering mechanism, causing runaway M1 hyper-activation and severe tissue stress propagation [91]. This supports that the ECS activation state directly contributes to determining whether a macrophage behaves as an engine of tissue destruction or an agent of metabolic resolution.

## 5. Adaptive Immune Modulation and Lymphocyte Reprogramming

Beyond its pronounced effects on the innate immune system, cannabinoid signaling exerts broad, highly coordinated regulatory control over adaptive immune responses [92]. T-cell activation, proliferation, and clonal expansion are heavily suppressed via cannabinoid-mediated inhibition of interleukin-2 production at the transcriptional level, alongside direct disruption of T-cell receptor (TCR) distal signaling cascades and proximal Janus kinase (JAK) and signal transducer and activator of transcription (STAT) pathways [92,93]. Furthermore, CB2 signaling skews the differentiation of naive CD4+ T cells away from the highly inflammatory T helper 1 (Th1) and Th17 lineages, favoring instead the generation of CD4+CD25+FoxP3+ regulatory T cells (Tregs) [94,95]. In the context of adaptive immunity, the therapeutic paradox manifests as a critical, structural trade-off between autoimmune tolerance and the maintenance of pathogenic immunosurveillance. This shift is highly effective at reinforcing immune tolerance and alleviating autoimmune disease states, but it severely impairs host antiviral and antitumor immunity, which depend entirely on robust, unhindered Th1 and cytotoxic T-lymphocyte responses [96]. In oncological microenvironments, this immunosuppressive influence can turn counterproductive. By limiting T-cell infiltration, recruiting myeloid-derived suppressor cells, and blunting local cytotoxic immune surveillance, cannabinoid exposure can reduce the therapeutic efficacy of contemporary immune checkpoint inhibitors and accelerate tumor progression in specific malignant niches [97].

B-cell function is similarly modulated. CB2 activation reduces B-lymphocyte proliferation, dampens antibody class switching, and attenuates terminal plasma cell differentiation, which can lead to reduced titers of antigen-specific immunoglobulins during an active infection [98,99]. Dendritic cells are also bound by this transcriptional paralysis. Under CB2 stimulation, maturing dendritic cells exhibit a marked failure to upregulate foundational costimulatory surface proteins such as Cluster of Differentiation 80 (CD80), CD86, and major histocompatibility complex (MHC) class II, severely slowing down their antigen-presentation capacity and delaying the generation of crucial adaptive activation loops required to handle novel systemic pathogen challenges [20].

## 6. Functional Immunological Trade-Offs Across Human Cell Systems

To understand the therapeutic paradox of the ECS, its effects must be analyzed across distinct human cell lineages, balancing tissue-protective benefits against host-defense vulnerabilities. In macrophages, the pathway primarily involves the suppression of cAMP, PKA, and NF-κB, coupled with the activation of the Nrf2 pathway [68,100]. During the tissue-protective phase, this configuration drives classical M1-to-M2 macrophage phenotype reprogramming, upregulates protective IL-10 and arginase-1, and restores mucosal iron balance via hepcidin and ferroportin-1 regulation [81,90]. However, during the immunosuppressive phase, this same molecular shift blunts the essential oxidative respiratory burst, reducing ROS and nitric oxide (NO) production, which severely impairs the localized phagocytic eradication of fungal and intracellular bacterial vectors [78,85]. As previously mentioned in speculative frameworks, this suppression may also restrict mitochondrial bioenergetic pathways, potentially locking the cell into an autophagic resolution state that clears pathogenic defense structures prematurely [61,65].

In T lymphocytes, the molecular mechanism centers on JAK/STAT inhibition alongside a distal TCR block [92]. The tissue-protective benefit of this mechanism is the strict limitation of pathologically overactivated Th1 and Th17 lineages, which expands FoxP3+ Tregs to alleviate systemic autoimmunity [94,95]. The corresponding vulnerability is a deep dampening of cell-mediated helper and cytotoxic adaptive immune responses, which compromises early antiviral and oncological immunosurveillance [96,97].

In B lymphocytes, G protein-mediated kinase downregulation controls the cellular response [98]. This provides tissue protection by dampening auto-reactive B-cell expansion pathways and curtailing the production of highly destructive, pathogenic auto-antibodies [20]. Conversely, the immunological trade-off involves the suppression of physiological antibody class switching, which limits the generation of antigen-specific protective immunoglobulins during initial pathogen encounters [99].

Dendritic cells undergo a maturation transcriptional blockade under cannabinoid influence [20]. The clinical benefit is a restricted upregulation of costimulatory surface markers such as CD80, CD86, and MHC class II, interrupting severe autoimmune propagation cascades [41]. The accompanying vulnerability is a profound hindrance of downstream antigen presentation to naive T lymphocytes, delaying crucial adaptive activation loops [21].

In the central nervous system (CNS), microglia under CB2 stimulation utilize NLRP3 inflammasome suppression as their primary regulatory node [101]. This rescues vulnerable neuroarchitecture from chronic reactive microgliosis and mitigates neurovascular stress propagation [6]. However, this protective mechanism attenuates native CNS pathogen surveillance and clearance dynamics, elevating the host’s vulnerability to neurotropic viral insults [38] [Table 1].

Importantly, the biological effects of cannabinoid signaling differ considerably across distinct tissues and organ systems, commanding a comprehensive understanding of highly tissue-specific functions [102]. Within the brain, the ECS exerts neuroprotective effects primarily by quenching reactive microgliosis and modulating excitotoxicity, establishing a robust defense against neurodegeneration [103]. Conversely, in the liver, the ECS presents a functional dichotomy: CB1 receptor activation actively promotes lipogenesis, fibrogenesis, and steatosis during chronic hepatic injury [104,105], while CB2 receptor signaling effectively counters these mechanisms by asserting potent anti-fibrotic and hepatoprotective effects [105,106]. In adipose tissue, CB1 activation is fundamentally linked to metabolic dysregulation, directly driving lipogenesis, insulin resistance, and the recruitment of pro-inflammatory macrophages, rendering the blockade of peripheral CB1 a valuable strategy for reversing obesity-associated metabolic disorders [107]. Furthermore, in the gastrointestinal tract, the ECS operates to maintain intestinal epithelial homeostasis, regulate gut motility, and actively suppress pathological mucosal inflammation, an effect chiefly mediated by CB2-driven attenuation of inflammatory bowel disease pathology [102,108]. Recognizing these pronounced tissue-specific differences provides a complete overview of the immunomodulatory role of the ECS and is essential to mitigate off-target effects when engineering advanced localized therapeutics [102,108].

## 7. Strategic Frameworks for Next-Generation Cannabinoid Therapeutics

The ECS functions as a key homeostatic regulator of immunity, balancing necessary inflammation with tissue preservation. Its potent anti-inflammatory effects are mechanistically inseparable from transient immunosuppression, reflecting an evolutionary design that prioritizes protection against self-inflicted damage over maximal immune aggression. The contemporary therapeutic landscape demands highly precise engineering frameworks that separate the tissue-protective resolution programs of the ECS from its host-defense-compromising vulnerabilities. Six primary strategic methodologies are suggested to achieve this equilibrium.

Temporally controlled administration of cannabinoid therapeutics may prove advantageous as short-pulse, highly synchronized interventions. In conditions such as acute sepsis or ischemia–reperfusion injuries, unselective early activation can be catastrophic because it blocks the initial M1 oxidative burst and leukocyte recruitment waves necessary to trap and clear local viral or bacterial vectors [26,87]. By delaying administration until the precise kinetic inflection point where pathogen loads have decreased, and the host response threatens to spill over into automated hyper-inflammation, synthetic CB2 agonists can be utilized purely to accelerate the resolution phase without truncating the initial host-defense spike.

In terms of biased signaling and pathway-selective allosteric ligands, the complex architecture of the CB2 receptor allows for the development of biased agonists or positive allosteric modulators that exhibit functional selectivity [29]. Instead of triggering the entire Gi/o macromolecular cascade—which globally suppresses cAMP and turns off vital NF-κB host defense targets—biased signaling systems can be tailored to preferentially engage specific downstream pathways. For example, a molecule engineered to selectively drive the β-γ-mediated Nrf2 and HO-1 antioxidant network while leaving basal adenylyl cyclase activity uninhibited may provide deep tissue protection and antioxidant buffering without inducing full lymphocyte or macrophage paralysis [68,70].

To interpret the intracellular mechanisms explored in theoretical models, next-generation frameworks might cautiously prioritize subcellular and organelle-specific endocannabinoid pharmacology. Given the hypothesis that endocannabinoid receptors localized on internal organellar membranes exert precise control over immune bioenergetics, calcium buffering, and macroautophagy [58,63,65], engineering therapeutics that selectively penetrate these compartments represents a speculative but promising frontier. Synthetic endocannabinoid scaffolds could theoretically be covalently coupled to lipophilic cations, such as triphenylphosphonium moieties, to exploit the negative transmembrane potential of the inner mitochondrial membrane [61,63]. If validated, this organelle-directed delivery could allow for localized modulation of mitochondrial adenylyl cyclase without engaging plasma membrane-bound receptors, hypothetically decoupling bioenergetic homeostasis from broad cytokine paralysis [21,61]. Similarly, designing endocannabinoids linked to endoplasmic reticulum-targeting peptide motifs is proposed to tune the unfolded protein response and intracellular calcium oscillations, mitigating tissue stress propagation while sparing outer immune surveillance mechanisms [58,64].

For smart targeted delivery systems, to prevent global, off-target suppression of the immune compartments within the bone marrow, spleen, and peripheral lymph nodes, cannabinoid molecules can be encapsulated within advanced, context-sensitive nanocarriers [28]. Nanoparticles can be surface-functionalized with antibodies or peptides that target ligands overexpressed exclusively on inflamed tissues, such as vascular cell adhesion molecule 1 (VCAM-1) in neurovascular tracking or specific integrins in localized colonic lesions [18,45]. These carriers can also be engineered with trigger mechanisms that release the cannabinoid payload only under specific environmental conditions, such as low extracellular pH environments typical of ischemic or highly glycolytic inflammatory foci, high ROS concentrations, or elevated local matrix metalloproteinase levels. This restriction confines CB2 activation directly to the site of pathological injury, preserving unhindered immune surveillance throughout the rest of the host organism.

Regarding rational combination therapies, cannabinoids could ultimately be integrated as adaptive adjuncts alongside traditional therapeutic agents to construct a balanced dual-force regimen [21]. In severe infectious or oncology settings, a CB2 agonist can be co-administered with narrow-spectrum antimicrobials, tumor-targeted cytotoxic therapies, or low-dose immunostimulatory cytokines, such as IFN-γ [96,99]. In this structural pairing, the antimicrobial or specialized oncology compound handles the objective eradication of the pathogen or malignant line, while the cannabinoid directly shields the surrounding parenchymal cells from the collateral, self-destructive bystander damage typically caused by the host’s own inflammatory response.

For biomarker-guided personalization, clinical deployment of ECS-modulating drugs requires strict patient stratification based on functional immunophenotypical signatures [10]. Patients should be dynamically monitored using multi-omic screening panels that track total plasma endocannabinoid profiles, leukocyte CB2 surface expression density, circulating macrophage polarization ratios, and local mucosal barrier breakdown indices [46]. A patient presenting with an over-activated, high-velocity inflammatory endotype with minimal infection risks represents an ideal candidate for deep cannabinoid-mediated resolution induction. Conversely, a patient showing signs of immune exhaustion or high occult viral or bacterial colonization would be flagged as a high-risk candidate for cannabinoid therapy, avoiding the introduction of dangerous immunosuppressive windows.

In terms of multi-target endocannabinoidome modulation, instead of using potent, fully synthetic external agonists that lock the receptor into prolonged activation, therapeutic strategies should shift toward tuning the indwelling metabolic machinery of the endocannabinoidome [36]. The application of use-dependent, specialized inhibitors of MAGL or FAAH allows for the highly localized preservation of endogenous 2-AG and AEA pools, ensuring that receptor engagement occurs in an autocrine fashion only within cells that are already actively undergoing inflammatory stress [31,35]. Furthermore, the synthesis of hybrid, multi-target divalent molecules—which combine a cannabinoid receptor modulating scaffold with an independent pro-resolving lipid mediator core, such as a resolvin or protectin domain—offers a promising avenue for synergistic cannabinoid therapeutic strategies [4].

## 8. Conclusions

The ECS represents a sophisticated evolutionary architecture designed to act as a modulator of immune balance, continually trading maximal host-defense aggression for immediate structural tissue preservation [30]. Its ability to downregulate hyper-acute, runaway inflammatory programs via the Gi/o, cAMP, PKA, and NF-κB axis [51,52] and upregulate protective, antioxidant-driven tissue repair mechanisms through the Nrf2 and HO-1 pathway [68] makes it a valuable pharmacological target for clinical medicine [11]. However, as defined by the therapeutic paradox outlined in this review, these resolution pathways fundamentally share structural biochemical nodes with essential host immunity mechanisms [26], and unselective or poorly timed intervention will inevitably result in a compromised state of antimicrobial readiness [99]. Furthermore, models relying on sub-cellular and organelle dynamics must be approached with caution, as they remain largely speculative and require robust in vivo validation.

By moving beyond broad cannabinoid receptor activation and adopting more precise engineering strategies—such as temporal control, biased allosteric signaling, cautious exploration of organelle targeting, localized nanocarrier delivery, and endocannabinoidome tuning—researchers can transform cannabinoids from broad immunosuppressants into targeted immunomodulatory therapies. Collectively, available evidence supports the ECS as an attractive immunomodulatory target. However, major questions remain regarding receptor context dependence, tissue specificity, timing of intervention, and the translational relevance of emerging intracellular signaling mechanisms. Addressing these questions will be essential before many of the conceptual strategies proposed here can be translated into clinical practice.

## Figures and Tables

**Table 1:** Immunological trade-offs and matrix of CB2 receptor activation across human cell systems.

Target System	Primary Pathways Involved	Tissue-Protective Phase Benefits	Immunosuppressive Phase Vulnerabilities
Macrophages	cAMP ↓, PKA ↓, NF-κB ↓, Nrf2 ↑	Drives classical M1-to-M2 phenotype reprogramming; upregulates IL-10 and Arginase-1; dampens systemic leukocyte rolling affinity.	Blunts essential oxidative respiratory bursts (ROS/NO); impairs localized phagocytic eradication of fungal and intracellular bacterial vectors.
T Lymphocytes	JAK/STAT inhibition, Distal TCR block	Limits pathologically overactivated Th1/Th17 lineages; induces expansion of FoxP3+ regulatory T cells to alleviate systemic autoimmunity.	Severely dampens cell-mediated helper and cytotoxic adaptive immune responses; compromises early antiviral and cellular immunosurveillance.
B Lymphocytes	G protein-mediated kinase downregulation	Dampens auto-reactive B-cell expansion pathways; curtails production of highly destructive, pathogenic auto-antibodies.	Suppresses physiological antibody class switching; limits generation of antigen-specific protective immunoglobulins during initial infection.
Dendritic Cells	Maturation transcriptional blockade	Restricts upregulation of costimulatory surfaces (CD80/CD86/MHC-II); interrupts severe autoimmune propagation cascades.	Hinders downstream antigen presentation to naive T lymphocytes, delaying crucial adaptive activation loops.
Microglia (CNS)	NLRP3 inflammasome suppression	Rescues vulnerable neuroarchitecture from chronic reactive microgliosis; mitigates severe neurovascular stress propagation.	Attenuates native central nervous system pathogen surveillance and clearance dynamics; elevates vulnerability to neurotropic viral insults.

Abbreviations: cAMP, cyclic adenosine monophosphate; CB2, cannabinoid receptor 2; CNS, central nervous system; IL-10, interleukin-10; JAK/STAT, Janus kinase/signal transducer and activator of transcription; MHC-II, major histocompatibility complex class II; NF-κB, nuclear factor kappa B; NLRP3, NLR family pyrin domain containing 3; NO, nitric oxide; Nrf2, nuclear factor erythroid 2-related factor 2; PKA, protein kinase A; ROS, reactive oxygen species; TCR, T-cell receptor; Th1/Th17, T helper 1/T helper 17.

## Data Availability

No new data were created or analyzed in this study. Data sharing is not applicable to this article.
